# The Progress of the Prevention and Treatment of Vitamin D to Tuberculosis

**DOI:** 10.3389/fnut.2022.873890

**Published:** 2022-05-17

**Authors:** Li Cai, Gaoming Wang, Peijun Zhang, Xinyi Hu, Hao Zhang, Fan Wang, Yeqing Tong

**Affiliations:** ^1^Wuhan Center for Disease Control and Prevention, Wuhan, China; ^2^School of Health Sciences, Wuhan University, Wuhan, China; ^3^East and West Lake District Center for Disease Control and Prevention, Wuhan, China; ^4^Hubei Center for Disease Control and Prevention, Wuhan, China; ^5^Global Study Institute, University of Geneva, Geneva, Switzerland; ^6^School of Public Health, Wuhan University of Science and Technology, Wuhan, China

**Keywords:** tuberculosis, prevention, treatment, vitamin D, association

## Abstract

The progressions of a number of lung diseases, including acute lung injury, cystic fibrosis, asthma, chronic obstructive pulmonary disease, pneumonia and tuberculosis (TB) are found to be highly associated with inflammatory responses. As a signaling nutrient, Vitamin D modulates the activities of dendritic cells, monocytes/macrophages, T and B cells, and tissue epithelial cells in the body to induce inflammatory responses and boost immune functions. Given the high prevalence of vitamin D deficiency among pulmonary insufficiency and inflammation-related cases, researchers indicated vitamin D supplementation could have a potential role in the prevention and treatment of lung disease, especially tuberculosis. In this paper, we reviewed published studies on the role of vitamin D in the prevention and treatment of tuberculosis. The paper identified vitamin D’s potential as an adjunctive therapy and demonstrated its safety so as to provide an impetus for further studies and clinical applications.

## Introduction

### The Metabolism of Vitamin D

Vitamin D (VitD) is a family of vitamins derived from cholesterol. Two major forms of VitD are vitamin D2 (VitD2) and vitamin D3 (VitD3), of which VitD2 is mainly found in plants and VitD3 is only found in animal-sourced food, such as fish oil, liver, egg yolk, butter, and dietary supplements (1). VitD2 differs from VitD3 by its additional methyl group and a double bond. Although different in structure, the two forms of VitD, when activated, exhibit similar responses in the body (2).

VitD3 is synthesized by skin tissue from 7-DHC after a dihydroxylation step. 7-dehydrocholesterol (7-DHC) is a vitamin precursor that is synthesized by cholesterol oxidation and then transferred to skin tissue and stored in keratinocytes and fibroblasts. After binding to a protein, it is transferred to the liver and metabolized to 25-hydroxyVitD by 25-hydroxylase, Vitamin D-binding protein (VDBP) is the major binding protein for VitD metabolites. It mainly plays three important roles in the physiological mechanisms of VitD: enlargement of the biological half-life of VitD (binding protects VitD from bio-degradation), limiting its access to target tissues and maintains plasma VitD levels through reabsorption in the kidneys. To be specific, 85–90% of the total circulating VitD metabolites are bound to VDBP, 10–15% are bound to albumin, and less than 1% remains free in the serum (3, 4). The protein activity in the bound form is affected, so only the unbound protein can exert its activity (4, 5). Thus, the binding of VitD to VDBP impairs its delivery to target cells.

In the form of D2 or D3, Vitamin D is converted to 25-hydroxy-D2 or 25-hydroxy-D3 after intestinal absorption in the form of D2 or D3, respectively. Next, it enters the circulatory system and combines with VDBP. In the renal tubules of the kidney, 25-hydroxyl D is hydroxylated by enzymes 1-a hydroxylase and 24-a hydroxylase to produce the active forms 1,25 dihydroxy-D and 24,25 dihydroxy-D and bind to the nuclear hormone receptor with high affinity under the regulation of phosphate, calcium, fibroblast growth factor, and parathyroid hormone. Finally, 24, 25-dihydroxyVitD is oxidized to form water-soluble metabolites, excreted *in vitro* by bile and urine (6, 7).

### The Pathogenesis of Tuberculosis

The immune response of the human host to Mycobacterium tuberculosis (MTB) is of special significance from disease onset to have pathophysiological outcomes to observed clinical outcomes (8). Tuberculosis (TB) is an infectious disease caused by MTB. It is mainly transmitted through inhaling tiny droplets from the cough or sneezes of an active infected patient. The bacteria then is activated into alveolar macrophages (9), thus forming early infection foci. The initial process of TB growth within macrophages results in the formation of TB foci with solid caseous necrosis in the center, which limits TB’s further replication. Caseous necrosis presents a cheese-like appearance. During this period, cellular immunity mediated by T cells and delayed hypersensitivity reactions develop, which have a decisive impact on the progression and prognosis of TB (10). Following the symbiotic stage, when bacillus keeps multiplying within the macrophages, macrophages accumulate and divide. In caseous necrosis, TB bacteria are capable of growing but not reproducing. Once liquefied, the necrosis provides an ideal breeding environment for bacteria. Thus, the caseous central site of fibrous necrotic foci is considered to be the main site for bacterial persistence.

## Subsections Relevant for the Subject

### The Mechanism of Vitamin D in the Treatment of Tuberculosis

Macrophage decreased while using a VitD-mediated immune response against TB bacteria and further reduced with the addition of cytokines IFN-g and CYP27B1 (macrophage promoter). In 2006, an increased expression of both vitamin D receptor (VDR) and CYP27B1 was detected by TLR2/1 by Liu et al. (11). This is due to the VDR transactivation of an antimicrobial peptide (cathelicidin, LL37) that can successively activate the intracellular PRR and NOD2 and ultimately activate the NF-KB. The VitD receptors have macrophages on the surface. The interaction between macrophages and Mycobacterium tuberculosis, through activation of receptors I and II, enhanced 1α-hydroxylase (CYP27B1), VDR and Catherine Xitin (VDR target organ) expression. Meanwhile, VitD induces the production of methyl glycol and β-Fenin 2 (antimicrobial peptide) to introduce monocytes, neutrophils, and T cells to the site of infection, playing an immunomodulatory role in the treatment of TB (12).

### The Current Status of Vitamin D in the Prevention and Treatment for Tuberculosis

Determinants of VitD status included age, race, skin color, and living habits. VitD deficiency is prevalent across countries. Such deficiency is widely found in latent and active tuberculosis patients. Researchers have made the below argument to illustrate the association between VitD and tuberculosis: VitD deficiency could aggravate the susceptibility and accelerate the progression of the disease (13, 14). Thereby, it is assumed that VitD supplements could play a role in the prevention and treatment of tuberculosis.

Studies demonstrated that VitD level is highly associated with the susceptibility of hosts to mycobacterium tuberculosis. Researchers enumerated three observations that could illustrate its association: first, cold winter with reduced VitD from sun exposure is the season of high tuberculosis incidence; second, people with specific demographic characteristics (e.g., children, the elderly, uremic patients, and Asian immigrants in the United Kingdom) are prone to have higher tuberculosis incidence rate and lower serum VitD level than the left groups (14–16). Hewison explained that VitD level is closely related to innate and adaptive immunity, which made those people susceptible to tuberculosis (17).

VitD can also be an adjuvant treatment for tuberculosis. The study by Zhang and Li found that VitD supplementation is beneficial to the conversion of TB and can significantly reduce the positive rate of sputum culture in patients and shorten the negative transfer time of sputum culture (15). Researchers also found that adequate VitD supplementation shortened the conversion time of sputum smear and sputum culture in patients (18, 19). Moreover, related investigations also demonstrated that VitD is capable of being an adjuvant treatment for multidrug-resistant tuberculosis (MDR-TB). Therefore, VitD deficiency may lead to the prolonged negative conversion time of sputum smear in patients with MDR-TB (20). Regarding the mechanism by which VitD supplementation works, Hewison (17) noted that VitD could facilitate the clearance of TB bacteria in the body.

**TABLE 1:** Main findings of vitamin D for tuberculosis prevention and treatment.

Conclusion	Argument	References
Vitamin D supplement prevent infection after exposure	Cold winter with less vitamin D supplement is the season of tuberculosis high incidence	(14)
	Populations with some demographic and clinical characteristics that affect vitamin D levels vary in incidence	(14–16)
	Serum vitamin D level is closely associated with immunity that could protect people from infection	(13, 17)
Vitamin D supplement could be an adjuvant treatment of tuberculosis	Vitamin D supplement facilitates the clearance of TB bacteria	(17)
	Vitamin D supplement facilitates sputum conversion rate and treatment completion rates of TB, even for MDR-TB or patients co-infected with HIV	(15, 18–20, 24, 25)
	Vitamin D supplement accelerates clinical and radiographic improvement	(19, 21, 23)
	Vitamin D supplement increase the overall cure rate	(22)

**FIGURE 1:**
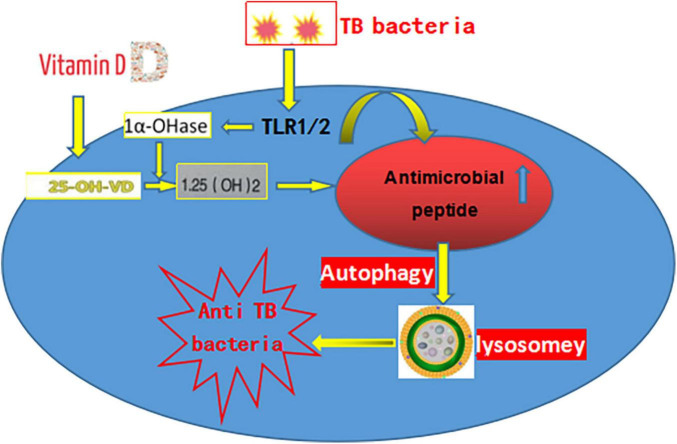
The pathogenesis of tuberculosis.

In addition to its contribution to shortening conversion time, VitD supplementation can also accelerate clinical and radiographic improvement for active TB patients. Through case-control studies, researchers found patients in the VitD supplementation group had greater clinical improvement. Significantly fewer patients had fever in the control group (19, 21). The study of Dai found that the patient’s serum 25-hydroxyvitamin D level was slightly related to the outcome of tuberculosis treatment (22). The higher the 25-hydroxyvitamin D concentration detected in the patient, the greater the cure rate. Sutaria et al. conducted a systematic review pertaining to case-control studies in this area to examine the potential of VitD. The study concluded that supplementation with VitD to tuberculosis patients can improve clinical outcomes (23). Jahnavi and Sudha conducted a case-control study for 100 patients undergoing anti-tubercular therapy in Anganwadi Centres of India. Researchers provided food supplements that can meet the daily intake target of vitamins and minerals for the experimental group. Patients who were randomly assigned to the control plan did not get any dietary plan or food supplements. After 3 months, patients in the experimental group reached significant higher sputum conversion rates and treatment completion rates (24). Moreover, by conducting a two-by-two factorial trial in Tanzania, Range et al. argued that vitamin and mineral supplementation could significantly increase the survival rate of sputum-positive patients co-infected with HIV (25).

### The Adverse Reactions of Vitamin D Treating Tuberculosis

In 1969, Brincourt proposed to use 15 mg (600,000IU) VitD to treat TB (26). In 1981, Gwinup et al. proposed to treat TB with 125 μg VitD2 for TB (27). During the same period, the study by Stern et al. (28) and Tjellesen et al. (29) used a high dose of VitD to treat TB, but there were still no adverse effects. In 2006, A study found that 250 μg of VitD per day for 6 weeks after the start of anti-TB treatment in Indonesia could clear TB bacteria more quickly from sputum. The imagings of patients with TB were also significantly improved (21). A cohort study of multiethnic patients with TB in the United Kingdom showed that a single oral dose of 2.5 mg of vitamin D2 in the treatment of TB has no adverse reactions such as hypercalcemia (30). In 2012, a randomized controlled trial showed that oral dose of 2.5 mg of vitamin D as an adjuvant treatment could shorten the sputum smear-negative rotation time in 95 treated patients with TB (31). Currently, that dose of vitamin D has not been reported to be applied to elderly patients with TB.

### Limitation

Although it has been confirmed by many researchers that VitD can be used as adjuvant therapy for tuberculosis, there are still limitations in the existing research. For clarity, so far, there is no consensus on the reference values that define VitD sufficiency and deficiency. Studies cited by this paper mostly take the reference value as the mean serum 25-hydroxyvitamin D concentration in the population of the research regions. Besides, whether vitamin concentration could be considered to be sufficient also varies for its different functions, such as for bone health or for immunity. In this regard, the optimal amount of VitD supplements for the prevention and treatment of TB remain unknown. Research breakthrough on the reference value is needed (32).

The significance of the association between VitD status and tuberculosis still left the direction of the causation relationship quite uncertain. On the one hand, TB patients with already low VitD levels may have lower VitD since the commencement of treatment (33, 34). On the other hand, VitD deficiency might be an important determinant for patients with latent tuberculosis to become active. Therefore, further studies that could explicate the mechanism of VitD in preventing and treating tuberculosis should be done.

## Discussion

### The Progress of Vitamin D in the Treatment and Prevention for Tuberculosis

The analysis from Huang et al. found that vitamin D level is positively associated with the CD4 + and CD8 + expression in the patient (35) and negatively associated with the patient’s condition, which may be related to the low epidemic ability and unbalanced immune regulation in T cell rabbits, and the susceptibility to TB may even aggravate the patient’s progress. The study shows that the number of reported TB in the elderly, aged 65 years old accounted for about 1/5 of total TB whether the diagnosis and medication compliance, or the prevention and treatment of elderly TB patients are relatively difficult (36). Age growth can lead to VitD metabolism disorders, coupled with less light time and eating, physiological decline of renal function, and reduced endogenous VitD synthesis, can result in a low concentration of blood 25-hydroxyvitamin D. At present, many elderly people with osteoporosis add calcium and vitamin D, indirectly enhance the physical fitness, has a positive effect to the prevention and treatment of TB for themselves.

A longitudinal study of patients with TB in Lahore, Pakistan, found that TB patients have severe vitamin D deficiency (37), but the Lahore region has an annual ultraviolet radiation intensity enough to induce VitD synthesis in the skin. The mean serum VitD concentration (27.3 nmol/L) was lower than the reported concentration (40.5 nmol/L) elsewhere, presenting many lung lesions and mostly bilateral lesions. Among the patients with presumed active TB, VitD deficiency preceded active TB. However, the effects of reverse causality or potential confounders cannot be excluded. Maintaining sufficient VitD levels in patients with TB may help to control the infection and activation of TB. Individuals can adjust their lifestyle and take appropriate vitamin D supplementation as a means of preventing and treating TB.

### Challenge and Outlook

Taken together, according to the results of preceding studies, Vit D plays an important role in the prevention and treatment of tuberculosis. For TB patients, it is recommended that clinicians should strengthen nutrition monitoring to improve the VitD level in the body of patients. This review is mainly to advise decision-makers to supply VitD as a preventive and adjuvant treatment for tuberculosis susceptible populations and patients. It is suggested to add VitD supplements to TB standard regimen, especially that of the elderly, as an important progress in the personalized treatment of TB.

## Author Contributions

YT, LC, and PZ: conceptualization. XH and GW: methodology. HZ and FW: resources. YT and XH: writing—original draft preparation. LC: writing—review and editing. HZ: visualization. XH: supervision. FW: project administration. YT: funding acquisition. All authors contributed to the article and approved the submitted version.

## Conflict of Interest

The authors declare that the research was conducted in the absence of any commercial or financial relationships that could be construed as a potential conflict of interest.

## Publisher’s Note

All claims expressed in this article are solely those of the authors and do not necessarily represent those of their affiliated organizations, or those of the publisher, the editors and the reviewers. Any product that may be evaluated in this article, or claim that may be made by its manufacturer, is not guaranteed or endorsed by the publisher.
